# Crowdsourced Indoor Positioning with Scalable WiFi Augmentation †

**DOI:** 10.3390/s23084095

**Published:** 2023-04-19

**Authors:** Yinhuan Dong, Guoxiong He, Tughrul Arslan, Yunjie Yang, Yingda Ma

**Affiliations:** School of Engineering, University of Edinburgh, Edinburgh EH8 9YL, UK

**Keywords:** indoor positioning, crowdsourcing, WiFi fingerprinting, machine learning, augmentation

## Abstract

In recent years, crowdsourcing approaches have been proposed to record the WiFi signals annotated with the location of the reference points (RPs) extracted from the trajectories of common users to reduce the burden of constructing a fingerprint (FP) database for indoor positioning. However, crowdsourced data is usually sensitive to crowd density. The positioning accuracy degrades in some areas due to a lack of FPs or visitors. To improve the positioning performance, this paper proposes a scalable WiFi FP augmentation method with two major modules: virtual reference point generation (VRPG) and spatial WiFi signal modeling (SWSM). A globally self-adaptive (GS) and a locally self-adaptive (LS) approach are proposed in VRPG to determine the potential unsurveyed RPs. A multivariate Gaussian process regression (MGPR) model is designed to estimate the joint distribution of all WiFi signals and predicts the signals on unsurveyed RPs to generate more FPs. Evaluations are conducted on an open-source crowdsourced WiFi FP dataset based on a multi-floor building. The results show that combining GS and MGPR can improve the positioning accuracy by 5% to 20% from the benchmark, but with halved computation complexity compared to the conventional augmentation approach. Moreover, combining LS and MGPR can sharply reduce 90% of the computation complexity against the conventional approach while still providing moderate improvement in positioning accuracy from the benchmark.

## 1. Introduction

Indoor positioning has attracted tremendous research interest in recent years. Although GPS has been served for years to provide location-based services in outdoor scenarios, it cannot provide highly reliable positioning accuracy in indoor environments due to signal attenuation and multi-path effects. Therefore, researchers have developed a variety of positioning techniques, such as using magnetic field [1,2,3], radio frequency identification (RFID) [4,5,6], and WiFi [7,8,9,10,11] to compensate for the shortage of GPS in indoor positioning.

Nowadays, most mobile communication devices, such as mobile phones, smartwatches, and laptops, are embedded with WiFi chips to detect WiFi signals in indoor environments. WiFi is widely adopted in many public and private areas to provide users with internet connections. Since no extra infrastructure is needed, fingerprinting based on WiFi received signal strength (RSS) has become the most prevalent method to solve the indoor positioning problem over the last decade [12]. This fingerprinting technique usually has two stages: online and offline stages [13,14,15]. The offline stage involves collecting data from wireless networks without actually being connected to them. This can be achieved using specialized equipment or software, such as wireless network scanners, that passively scan for nearby networks and collect data on their RSS value, SSID, and MAC address. Such data are usually annotated with the location where it was acquired, namely WiFi fingerprints (FPs). A radio map is then constructed based on the WiFi FPs to be used to identify the location of a device or track its movements. In the online stage, WiFi devices actively transmit data over the network, and their identifying information can be collected in real time by the user. The online data is compared to the FPs on the radio map to determine the most likely location where the signal was acquired.

A critical factor that affects the positioning accuracy using WiFi fingerprinting is the number of FPs on the radio map. Volunteers are asked to collect WiFi data at hundreds of reference points (RPs) in the investigated indoor region to obtain sufficient FPs. Such a site survey is usually time-consuming and labor-intensive [16]. The study from Wang et al. [17] mentions that it took about 10 h for two graduate students to take FPs at 150RPs of an area of 281 m^2^. It is even worse when considering a multi-floor building scenario, such as an airport or shopping mall.

Therefore, a crowdsourcing approach [18,19,20] has been proposed to collect WiFi data and record the location by utilizing the trajectories of common users. With the inertial sensors and WiFi cards in users’ smartphones, the WiFi RSS FPs can be collected and annotated by their location and movements [21]. For example, HimLoc is specifically tailored for smartphone users and makes use of commonly available sensors such as the compass, accelerometer, and WiFi card. Its functionality is based on the idea of a crowdsourced WiFi training set, which is generated by the movements of people within the building [22]. LiFS [23] is a system that has been developed and is based on the sensors in mobile phones and user movement to create a radio map of a building. Unlike traditional radio-based solutions, it does not require site surveys and uses off-the-shelf WiFi infrastructure, making it easy to deploy with minimal human intervention. Even without site surveys, LiFS achieves accuracy that is comparable to previous approaches. However, relying on crowdsourced data also brings some challenges. For example, it is expected that the data amount will be very low at the beginning stage of deploying a crowdsourced indoor positioning system in a certain building due to a limited number of users. Such data is not enough to cover the entire indoor environment to provide an accurate positioning service. Furthermore, the crowdsourced data cannot guarantee to be updated everywhere as time changes though more user contributions are involved as the user number grows. This is attributed to low positioning accuracy in some areas as they do not have as many visitors as others.

This paper proposes a scalable WiFi FP augmentation method for 3D crowdsourced indoor positioning systems in large, complex indoor environments. The contributions are summarized as follows:

- We propose a WiFi FP augmentation method for 3D crowdsourced indoor positioning systems. The proposed method can generate effective WiFi FPs in unsurveyed locations to improve positioning accuracy.
- Two self-adaptive virtual RP generation (VRPG) approaches are designed based on and beyond the conventional approach in determining the virtual RPs of the new FPs.
- A multivariate Gaussian process regression (MGPR)-based spatial WiFi signal modeling (SWSM) algorithm is designed to model the distribution of all WiFi FPs and predict the signals on virtual RPs in a 3D environment.
- Experiments on an open 3D public dataset are conducted to comprehensively evaluate the positioning accuracy, floor identification accuracy, and computation complexity of the proposed augmentation method.

## 2. Problem Statement and Motivations

He et al. introduce a technique for creating a radio map with FPs based on the radio propagation model [24]. However, the radio model can be easily influenced by environmental changes, and the location of each AP is needed. Similarly, Jun et al. [25] present an indoor positioning system named AP-Sequence that aims to reduce the human effort in FP map creation with the location of each AP as a prior. Rather than using the propagation model, Sinhua et al. [26,27] propose to use linear interpolation to augment the WiFi RSS to generate more training data. Besides the linear interpolation method, Sun et al. [28] proposed using Gaussian process regression (GPR) to model the spatial distribution of the RSS of each AP and predict the RSS values of each AP on unsurveyed locations to generate new FPs. Nevertheless, such a method needs to create multiple GPR models for all APs in the target area. Since the signal distribution of each AP is different in the complex indoor environment, tuning all GPR models is inefficient and inappropriate for large indoor spaces with a large number of APs.

Through the literature analysis, one problem of the existing augmentation methods is scalability. Either using linear interpolation or pass loss model (propagation model) cannot well predict the signal distribution in different indoor environments. The recent machine learning-based approaches use regression models to model each AP’s signal distribution in the targeted indoor area. Although such methods have better generalization ability, they are not scalable and rational in practice. Usually, a WiFi RSS FP dataset for a building with multiple floors contains hundreds of APs. For example, an open-source dataset published in the EU Zenodo repository [29] contains more than 900 different MAC addresses in a five-floor building. Building and tuning the regression model for each AP for the entire building is highly time-consuming. Due to a lack of scalability, all the aforementioned methods were designed and evaluated in small 2D environments, such as a lab, an office, or a corridor associated with multiple rooms. It is doubtful whether such methods could still maintain the performance considering a large complex environment.

Furthermore, where to generate the new FPs should also be focused on when augmenting the WiFi FPs dataset. Most of the literature adopts a simple grid-based approach [30] that assumes the rectangle shape of a certain area according to the farthest points from the training RPs. However, such an approach does not consider the distribution of the RPs and will generate many non-necessary RPs that do not contribute to the positioning phase. The predicted new FPs on such non-necessary RPs will not improve the positioning performance but increase the computation complexity of the system.

Therefore, to solve the aforementioned two major problems, this paper proposes a novel WiFi FP augmentation method to improve positioning performance with higher scalability and lower computation complexity for 3D crowdsourced indoor positioning systems. The details are elaborated in the next section.

## 3. Methodology

### 3.1. Framework

**Figure 1:**
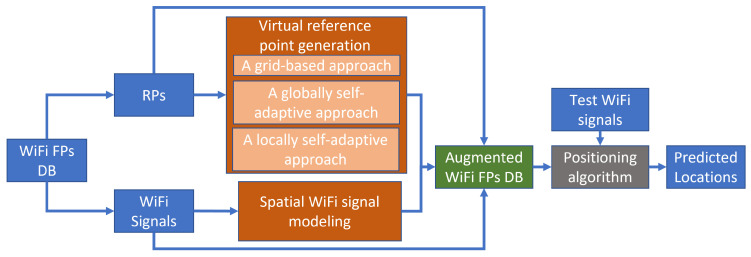
Framework of the proposed WiFi FP augmentation method.

As shown in Figure 1, the proposed method is mainly constructed of two elements: virtual reference point generation (VRPG) and spatial WiFi signal modeling (SWSM). Three VRPG approaches (two of which are proposed in this study) are developed to generate virtual RPs, and the SWSM is designed to model the WiFi FPs’ distribution to generate new WiFi signals on the virtual RPs to augment the dataset. The details are as follows.

### 3.2. Virtual Reference Point Generation

Each WiFi FP is composed of an RSS vector $f=[RSS_{1},RSS_{2},\ldots ,RSS_{M}]$ (*M* is the total number of detected APs in the entire dataset) and a location vector c=[x,y,z]. Considering a 3D coordinate system, *x*, *y*, and *z* stand for the coordinate along *x*, *y*, and *z*-axis (in meters). To obtain more WiFi FPs for positioning, we first stipulate a strategy to determine the locations where they should be generated. In this study, based on the grid-based (GB) approach [30] that was mentioned in the previous section, we propose two novel self-adaptive approaches: a globally self-adaptive (GS) approach and a locally self-adaptive (LS) approach.

#### 3.2.1. A Grid-Based Approach

Before introducing the two proposed approaches, we first introduce the simple and well-adopted GB approach. For a certain floor at *h* m, such an area can be simply described as a rectangle restrained by the maximum and minimum value of the coordinates of all the RPs along *x* and *y*-axes on the same floor, so that the four vertexes of such rectangle region are $\left(x_{min},y_{min},h\right)$, $\left(x_{max},y_{min},h\right)$, $\left(x_{min},y_{max},h\right)$ and $\left(x_{max},y_{max},h\right)$. We define a virtual RP in the targeted region as $c^{*}=\left[x_{i}^{*},y_{j}^{*},h\right]$. The entire region is partitioned into multiple 1 × 1 m^2^ grids along *x* and *y* axes. The coordinates of the virtual RPs are represented by the locations of the vertexes of each grid. So that:

(1) $$x_{i}^{*}=x_{min},x_{min}+1,x_{min}+2,\ldots x_{max}$$

(2) $$y_{j}^{*}=y_{min},y_{min}+1,y_{min}+2,\ldots y_{max}$$

hence, we can obtain *t* virtual RPs, where *t* can be calculated by:

(3) $$t=\left(x_{max}-x_{min}+1\right)\left(y_{max}-y_{min}+1\right)$$

**Figure 2:**
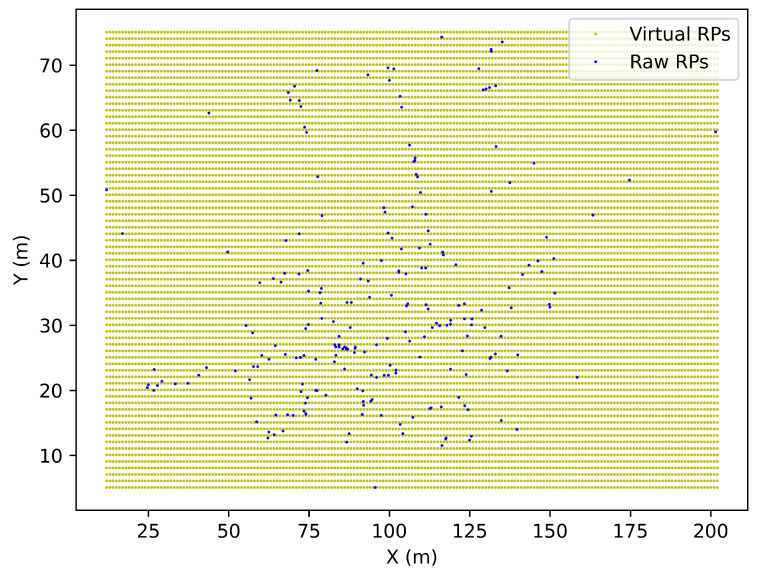
An example of applying the grid-based approach to a specific floor.

One example of applying the GB approach to a specific floor is shown in Figure 2.

#### 3.2.2. A Globally Self-Adaptive Approach

Beyond the GB approach, we design a new GS approach to reduce the non-necessary virtual RPs outside the targeted region.

The GS approach first generates the virtual RPs the same as the GB approach. Then, Graham’s scan [31] is used to detect the convex hull of the targeted region. Considering detecting the convex hull in a 2D environment on a floor, an initial RP with the lowest *y*-coordinate is selected. Then, the rest of the RPs are sorted in increasing order of the angle calculated between them and the initial RP along the *z*-axis. For the RPs with the same angle, only the farthest RP is kept (Euclidean distance is used to calculate the distance in this study). Then, we iterate the ordered RPs and calculate the angle formed by the three sequential RPs: previous RP, current RP, and next RP. The three RPs are kept if the angle is counterclockwise, or the current RP is dropped. Once the iteration finishes, the combination of the left RPs describes the convex hull of the targeted region, and only the virtual RPs that are inside the convex hull are kept.

To further remove the non-necessary virtual RPs, the virtual reference points are divided into many sub-areas by the mean-shift clustering algorithm. The mean-shift algorithm is a density-based unsupervised machine learning algorithm [32]. It senses changes in data density and updates the centroid by computing the average shift vector over a given centroid at an arbitrary sample RP. Given a set of data $x_{i},i=1,2,...,n$ on a *d*-dimensional space $R^{d}$, the mean shift vector of x can be expressed by:

(4) $$m_{h}\left(x\right)=\frac{\sum_{i=1}^{n}x_{i}G\left({∥\frac{x-x_{i}}{h}∥}^{2}\right)}{\sum_{i=1}^{n}G\left({∥\frac{x-x_{i}}{h}∥}^{2}\right)}-x$$

where $G\left({∥\frac{x-x_{i}}{h}∥}^{2}\right)$ is the kernel function and *h* is the bandwidth (h=10 in this study). The principle of mean shift is successively calculate the mean shift vector $m_{h}\left(x\right)$ and update the new x ($x=x+m_{h}$) until convergence.

**Figure 3:**
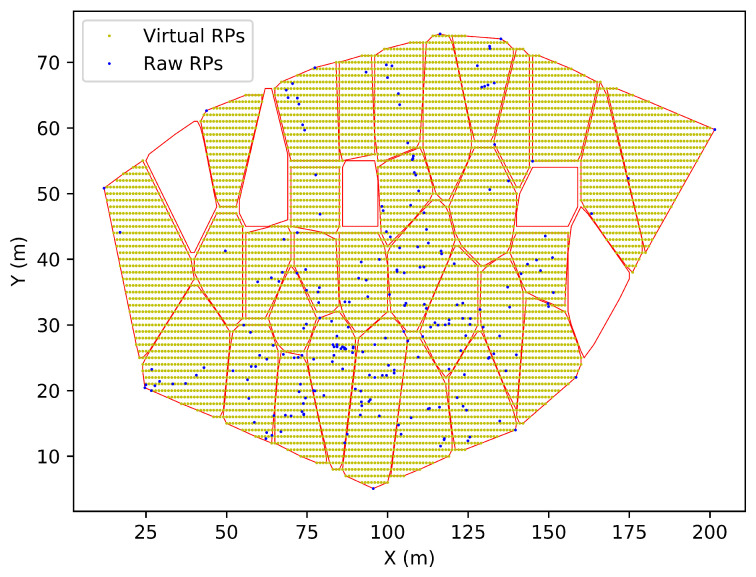
An example of applying the globally self-adaptive approach to a specific floor.

The RPs eventually converge to the same centroid and are clustered in the same sub-areas. The virtual RPs in the sub-areas with no raw RPs are removed. One example of applying the GS approach to a specific floor is shown in Figure 3.

#### 3.2.3. A Locally Self-Adaptive Approach

**Figure 4:**
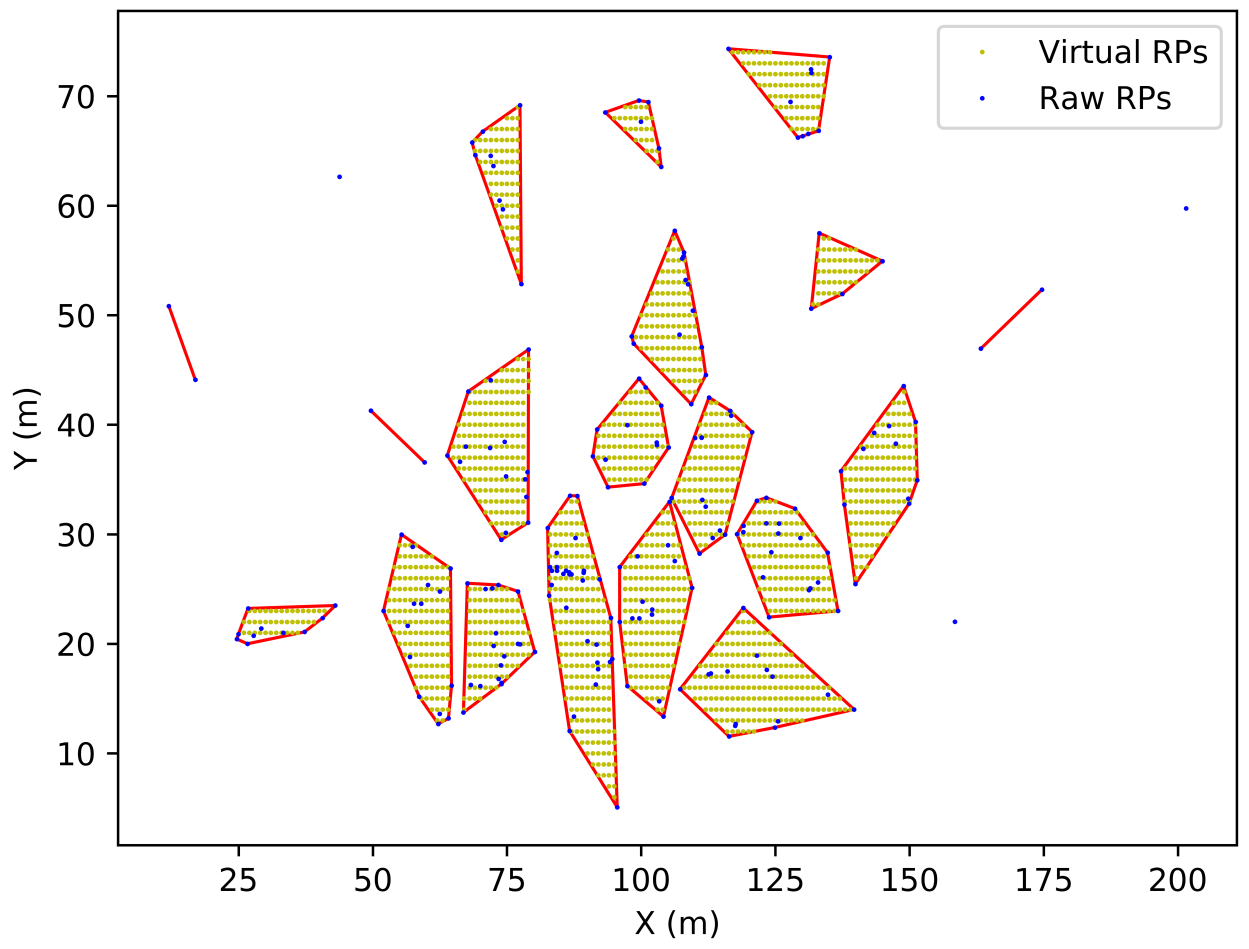
An example of applying the locally self-adaptive approach to a specific floor.

Different from the previous two approaches, we also propose an LS approach that focuses on exploring the local distribution of the raw RPs and detecting where the virtual RPs should be generated. For a targeted region, the local approach first uses a mean-shift cluster algorithm to separate the region into several sub-areas from the training samples. Only sub-areas with three points and more are processed by Graham’s scan method to recognize their convex hulls. A similar scheme is adopted to generate virtual RPs in each sub-area. One example of applying the LS approach to a specific floor is shown in Figure 4.

### 3.3. Spatial WiFi Signal Modeling

In this subsection, we present how we model the distributions of the spatial WiFi signal in a 3D environment to estimate the RSS values on the generated virtual RPs. For all RSS vectors $\left\{f_{1},f_{2},\ldots ,f_{N}\right\}$ in F, and $\left\{c_{1},c_{2},\ldots ,c_{N}\right\}$ in C, the mapping between the locations and the RSS vectors can be expressed by:

(5) F=λ(C)+η

where η represents the independent and identically distributed Gaussian noise with zero mean and variance, which can be denoted by $\eta ∼N(0,\sigma_{n}^{2})$.

To solve the mapping problem, we assume that all RSS vectors in the investigated indoor area obey a multivariate Gaussian process of multiple high-dimensional joint Gaussian distributions. Therefore, such a Gaussian process can be represented by the mean function m(C) and covariance function $k(c_{i},c_{j})$, as shown in the following equation:

(6) λ(C)∼GP(m(C),k(C))

(7) m(C)=E[f(C)]

(8) $$k\left(c_{i},c_{j}\right)=E\left[\left(f\left(c_{i}\right)-m\left(c_{i}\right)\right)\left(f\left(c_{i}\right)-m\left(c_{j}\right)\right)\right]$$

where E(·) denotes the expectation operator. Therefore, the covariance matrix K can be expressed by:

(9) $$K=\left[\begin{array}{cccc} k(c_{1},c_{1}) & k(c_{1},c_{2}) & \cdots & k(c_{1},c_{N})\\ k(c_{2},c_{1}) & k(c_{2},c_{2}) & \cdots & k(c_{2},c_{N})\\ \vdots & \vdots & \ddots & \vdots \\ k(c_{N},c_{1}) & k(c_{N},c_{2}) & \cdots & k(c_{N},c_{N}) \end{array}\right]$$

where *N* denotes the total number of WiFi FPs (or the total number of RPs).

Through the grid-based RP algorithm mentioned in the previous subsection, we obtain $N^{*}$ potential RPs $C^{*}$. This means that we have $N^{*}$ new WiFi RSS vectors (FPs) $F^{*}$ to be inferred. The RSS vectors F in the original dataset and RSS vectors $F^{*}$ to be inferred should also follow a joint multivariate Gaussian distribution, which can be stated by the following equation:

(10) $$\begin{array}{ccc} \; & \left[\begin{array}{c} F\\ F^{*} \end{array}\right]∼ & N\left(\begin{array}{c} \left[\begin{array}{c} m(C)\\ m(C^{*}) \end{array}\right],\left[\begin{array}{cc} K{\left(C,C\right)}_{N\times N} & K{\left(C,C^{*}\right)}_{N\times N^{*}}\\ K{\left(C^{*},C\right)}_{N^{*}\times N} & K{\left(C^{*},C^{*}\right)}_{N^{*}\times N^{*}} \end{array}\right] \end{array}\right) \end{array}$$

The posterior distribution $p(F^{*}|F)$ can then be expressed as:

(11) $$\begin{array}{c} f^{*}|f∼N\left(K\left(C^{*},C\right)K{\left(C^{*},C\right)}^{-1}F\right),\\ K\left(C^{*},C^{*}\right)-K\left(C^{*},C\right)K{\left(C^{*},C\right)}^{-1}K\left(C,C^{*}\right)) \end{array}$$

Hence, the posterior mean and covariance of the observed RSS vectors can be computed to obtain a model to predict the new RSS vectors on the unsurveyed potential RPs.

In addition, the covariance function $k\left(c_{i},c_{j}\right)$ (also called kernel function) plays a vital role in a Gaussian process to denote the relation between the RSS vectors and the corresponding RPs. One popular kernel function is the squared exponential kernel, which assumes that the process of the system is very smooth [33]. This is not suitable to describe the relationship among the high-dimensional RSS vectors in the large complex indoor scenario in our case. In [28], a mixture of Matern and Rational Quadratic (RQ) kernels performs the best in capturing the variation of RSS values in comparison with other kernels. The Matern kernel is defined as:

(12) $$\begin{array}{ccc} k_{Matern} & \left(c_{i},c_{j}\right)=\; & \frac{1}{\Gamma \left(\epsilon \right)2^{\epsilon -1}}{\left(\frac{\sqrt{2\epsilon }}{l}d\left(c_{i},c_{j}\right)\right)}^{\epsilon }K_{\epsilon }\left(\frac{\sqrt{2\epsilon }}{l}d\left(c_{i},c_{j}\right)\right) \end{array}$$

where ϵ denotes the smoothness of the function; *l* is the length scale; $K_{\epsilon }\left(\cdot \right)$ and Γ(·) are the gamma function and the modified Bessel function [33], respectively; *d* is the Euclidean distance which can be calculated by:

(13) $$d=\sqrt{{\left(c_{i}-c_{j}\right)}^{T}\left(c_{i}-c_{j}\right)}$$

The Rational Quadratic kernel can be described as:

(14) $$k_{RQ}\left(c_{i},c_{j}\right)={\left(1+\frac{d{\left(c_{i},c_{j}\right)}^{2}}{2\alpha l^{2}}\right)}^{-\alpha }$$

where α stands for the shape parameter. Therefore, the mixed kernel in our case is designed as follows:

(15) $$k_{mixed}=\mu *k_{Matern}+\nu *k_{RQ}$$

where μ and ν are the weighting parameters (initially set to 0.5). All the above-mentioned hyperparameters can be optimized by minimizing the negative logarithmic marginal likelihood.

After the MGPR model has been fine-tuned, it can forecast the RSS values for virtual RPs produced by the VRPG approaches, with the predicted RSS values presented in the same format as the training data. The predicted RSS values are then used to create new WiFi FPs, which are annotated with the location of the virtual RPs.

## 4. Preliminaries

### 4.1. Dataset

We evaluate the proposed WiFi FP augmentation method on a long-term public benchmark open-source WiFi fingerprinting dataset [29]. The data was collected from a five-floor University building (about 22,570 m^2^) in Tampere, Finland. The height of each floor is 0, 3.7, 7.4, 11.1, and 14.8 m, respectively. During the data collection stage, data was collected using twenty-one distinct Android devices, which were utilized by various individuals and placed in different orientations. In some cases, multiple people used the same device to replicate a scenario where data was crowdsourced.

The data is stored in CSV format and consists of four separate files: Coordinate, RSS, Date, and Device. Each of these files contains both training and test data and has multiple rows corresponding to the number of measurements recorded.

As listed in Table 1, 4648 FPs were collected, where 15% (697 FPs) were obtained for training and 85% (3951 FPs) for testing. A total of 992 MAC addresses (APs) were detected. The RSS value from non-detected APs in each FP was set to +100 by default in the dataset.

### 4.2. Data Pre-Processing

The RSS values from non-detected APs were set to −110 dBm before any analysis and experiments in both training and test sets. All RSS values were normalized by the maximum and minimum values before training the MGPR model. The 3D coordinates were also normalized by the maximum and minimum values along each training axes. The equation for normalization is shown below:

(16) $$m_{i}^{norm}=\frac{m_{i}-m^{\min}}{m^{\max}-m^{\min}}$$

where one sample $m_{i}$ is normalized to $m_{i}^{norm}$ by the maximum value $m^{\max}$ and minimum value $m^{\min}$.

### 4.3. Positioning Algorithm

This study did not propose a new positioning algorithm but applied a widely adopted weighted K-nearest neighbors algorithm (WKNN) to predict the locations. WKNN aims to find the *k* FPs in the training set with the nearest weighted distance to the test sample. The WKNN algorithm in this study was implemented using Scikit-learn [34]. The distance metric was set to Euclidean distance, and *k* was 3.

### 4.4. Training of the MGPR Model

**Figure 5:**
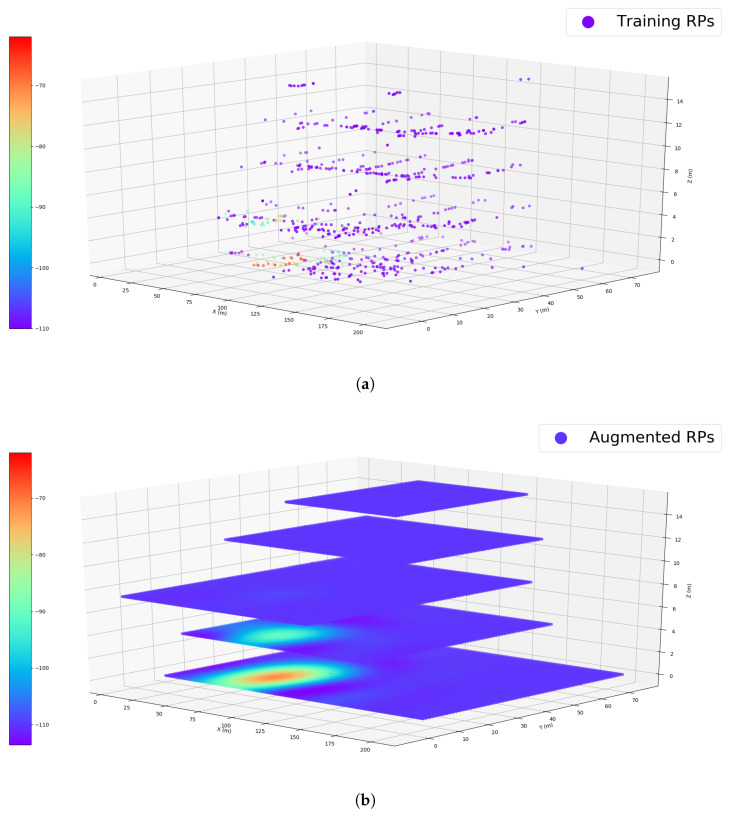
3D visualization of the heat map of AP 250 in the targeted building: (**a**) raw training data; (**b**) augmented training data (associated with GB approach).

To visualize the training results, we plot the heat maps over the multiple floors to show the generalization ability of the model. Figure 5 gives an example of comparing the WiFi signal distribution of one single AP (No.250) in the 3D environment. We can see from the figure that the well-trained model can predict the WiFi RSS values on the unsurveyed locations following a certain distribution in the 3D space. Although the majority of the observations of this AP are on the ground floor, the model can make informative predictions near the observations on both the same floor and the floor above.

## 5. Experiments and Evaluations

### 5.1. Experimental Settings

**Figure 6:**
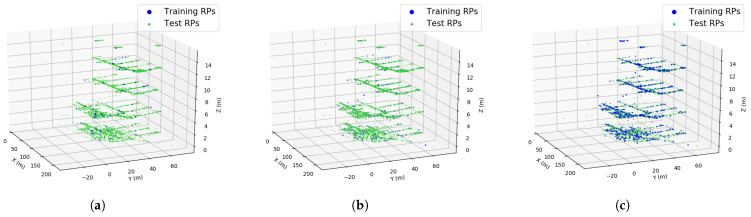
3D visualization of the partial training data and the test data: (**a**) sampling ratio = 10%; (**b**) sampling ratio = 50%; (**c**) sampling ratio = 100%.

In this experiment, we randomly selected a portion of the training data from the original training set with different percentages (from 10% to 100%). This was to simulate the growth of the training data coverage in the entire building. As the crowdsourced indoor positioning systems employ users’ contributions to construct the training set, the training data amount and its coverage are expected to be very limited at the beginning stage due to few users. As time goes by, the training set is expected to grow and finally cover the entire building as more and more users and their contributions are involved. It can be seen from Figure 6 that the coverage of the partial training data is very limited when the sampling ratio is very low. Most of the areas are not surveyed. As the sampling ratio increases, the training data grows and finally covers the entire building.

We trained the proposed MPGR model with such partial training data and predicted new RSS values on the virtual RPs from different VRPG approaches to generate new FPs. The new FPs and the partial training FPs were mixed into a new augmented dataset for positioning.

### 5.2. Performance Evaluations

In this subsection, we evaluate the performance of the proposed WiFi FP augmentation method through three aspects, including positioning accuracy, floor identification accuracy, and computation complexity.

#### 5.2.1. 3D Positioning Accuracy

**Figure 7:**
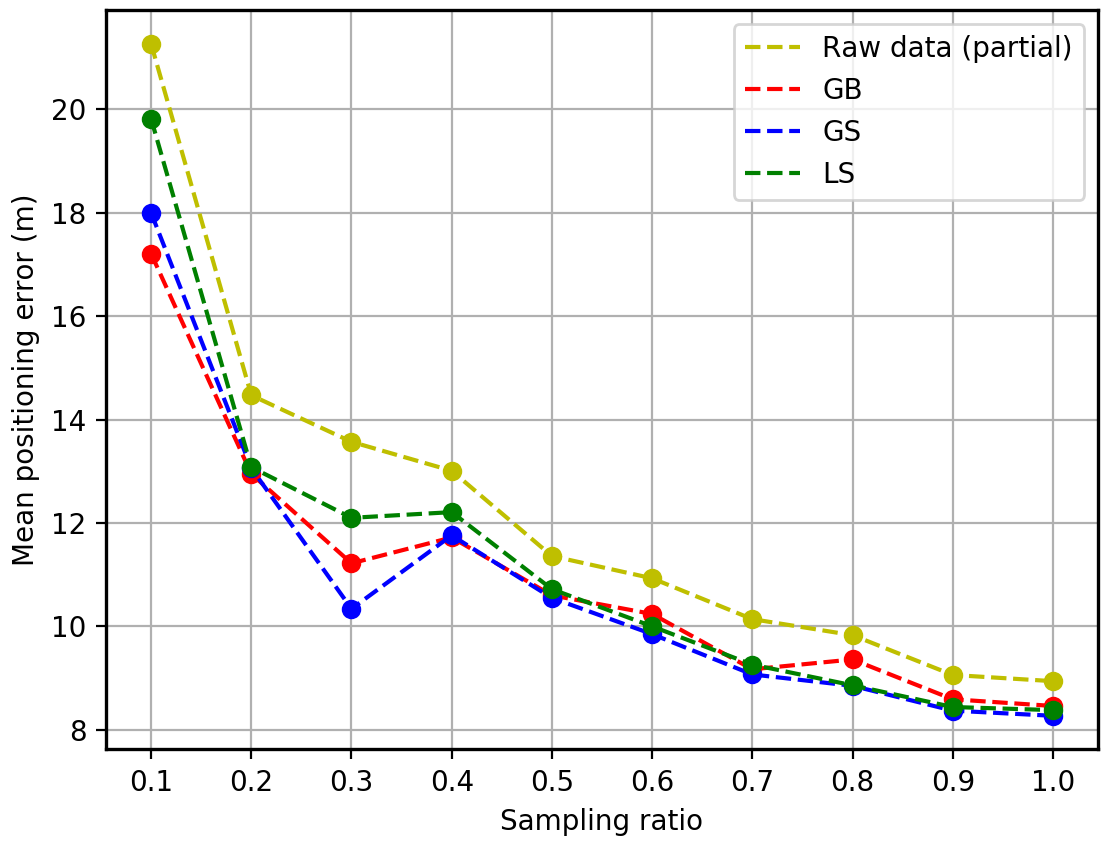
Mean positioning error of using different percentages of the training set.

The 3D positioning accuracy is calculated from the mean of the absolute positioning error between all predictions and the ground-truth coordinates. As shown in Figure 7, the positioning error decreases sharply at the beginning as the sampling ratio increases from 10% to 20%. Then, it declines steadily as more training FPs are involved. The positioning error with the augmented dataset using the GB, the GS approach, and the LS approach shows a similar trend as the partial training data but less error at each stage. We can observe from the figure that although there is a slight fluctuation, augmented data can always provide better positioning accuracy than raw data.

We can also observe from the figure that the approaches with augmented data show significant improvement (10–20%) at the beginning stage when only 40% of raw training data are used. However, the improvement decreases steadily when more raw training data is involved. This is because of the saturation of raw training data. Although the new FPs generated by the MGPR model can complement the limited number of raw training data at the beginning, the contribution of such new FPs weakens as the coverage of the raw training data grows.

Moreover, we can see some fluctuations in the improvement of positioning accuracy. This is because the distribution of raw training data is usually uneven, which is attributed to different positioning accuracy in different areas. Such a situation is consistent with the real situation that more users may visit some areas in the building, which provides more informative data and higher positioning accuracy. This motivates us to conduct the following experiment on region-based augmentation.

In addition, among the three approaches of using the augmented dataset, it can be seen from Table 2 that the LS approach usually provides a worse accuracy than the other two when the sampling ratio is lower than 50%. This is because such a low number of training data cannot cover most of the indoor environment. Hence, the augmented data from the sub-areas partitioned directly from the training data through the local approach cannot significantly improve the positioning accuracy. However, when the sampling ratio is higher than 50%, the positioning accuracy is better than the GB approach. Due to the higher data coverage in the indoor region, the LS approach can estimate the penitential infrastructure/floor plan of the building to generate more effective RPs, which can enhance the positioning performance.

Oppositely, the GS approach shows the best performance in most of the cases (7 out of 10) in providing higher positioning accuracy. This is because the GS approach augments the indoor environment by first detecting the boundary of the entire training data per floor. Although the training data cannot densely cover the entire region, the convex hull of the training data can describe the majority of the floor plan. Therefore, more virtual points can be generated for positioning.

#### 5.2.2. Floor Identification Accuracy

**Figure 8:**
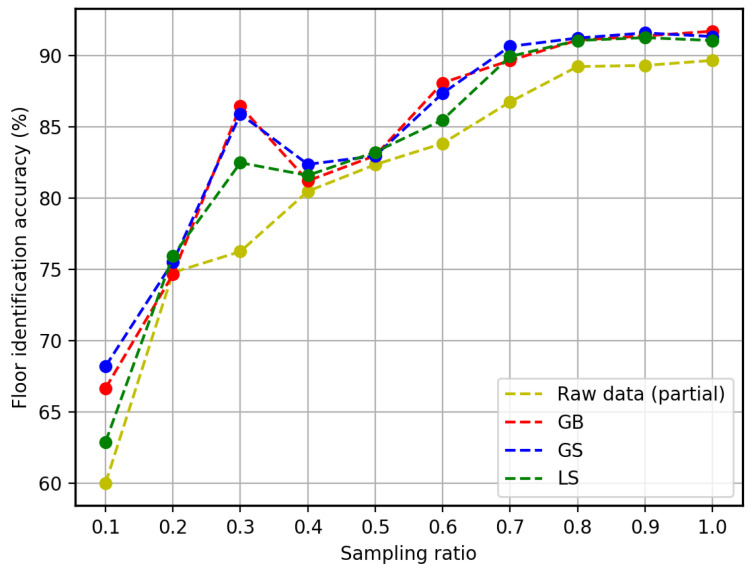
Floor identification accuracy of using different percentages of the training set.

Specifically, we calculate floor identification accuracy by using different approaches. The floor identification accuracy is calculated by the ratio of the number of cases predicted correctly and the total number of samples. It can be seen from Figure 8 that the approaches with augmented data can always provide a higher identification accuracy than only using the raw data, which is similar to the 3D positioning accuracy. As listed in Table 3, the GS approach can enhance floor identification accuracy more than the other two approaches (5 out of 10). It can significantly improve the accuracy (around 10%) even if there is little training data (less than 40%). While the performance of the LS approach is between the GB and the GS approach. Although it can improve the floor identification accuracy in all cases against using the raw data, the improvement is always limited to around 2%.

#### 5.2.3. Computation Complexity in Positioning Phase

In this part, we investigate the contribution of different VRPG approaches to the computation complexity of the final positioning algorithm. Given the final positioning algorithm of WKNN, the computation complexity of such an algorithm is usually proportional to the number of training samples, which can be calculated as:

(17) O[DN]

where *N* denotes the total number of training samples and *D* represents the dimensionality of each sample. In this study, each training sample is annotated with an RP. Therefore, the computation complexity of different augmentation approaches is mainly affected by the total number of RPs (mix of training RPs and virtual RPs).

**Figure 9:**
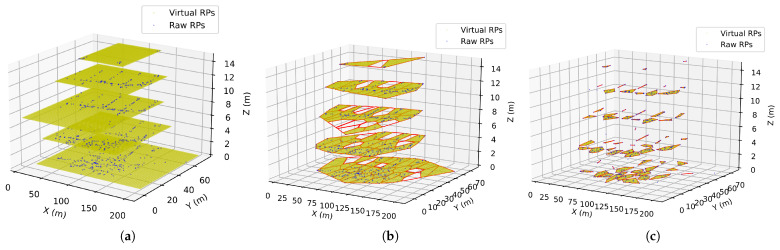
3D visualization of the augmented data from different approaches: (**a**) the grid-based approach; (**b**) the globally self-adaptive approach; (**c**) the locally self-adaptive approach.

As the numbers of total RPs in all cases are listed in Table 4, the GB approach always uses the largest amount of RPs, which leads to higher computation than the other two approaches. Compared to the GS approach, the computation complexity of GB is usually doubled. This is because the GB approach generates the virtual points by filling the indoor region with grids partitioned from a rectangle boundary by the farthest RPs in the training data. Although such an approach can cover all possible indoor areas even when the data amount is insufficient, it also generates a lot of virtual points that do not belong to the investigated indoor areas. As an example is shown in Figure 9, most of the virtual points are outside of the real floor plan, which does not contribute to improving the positioning performance but degrades the computation complexity. Beyond the GB approach, the proposed GS approach detects the convex hull and conducts the information density analysis, which eliminates most of the areas that are out of the investigated region to reduce the computation complexity.

**Figure 10:**
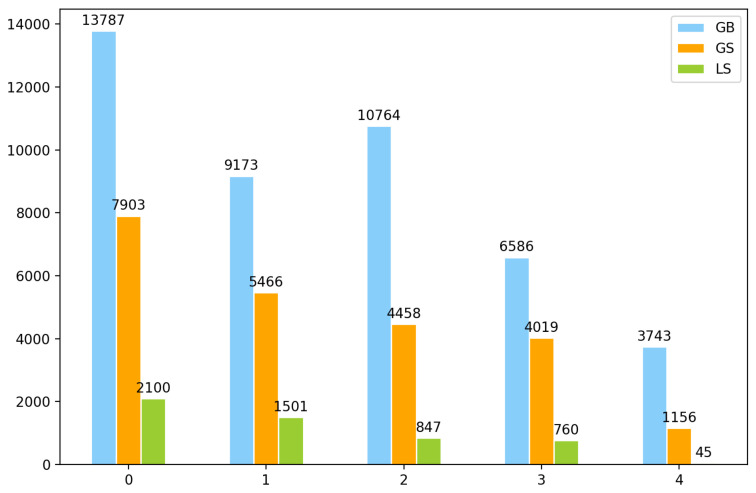
The number of RPs in the different floors (when sampling ratio = 100%).

Other than the GB and GS approaches, the proposed LS approach shows extremely low computation complexity. Such an approach can provide reliable improvement in positioning performance with a low computation complexity when there is sufficient data. It can be seen from Figure 10 that LS uses no more than 10% of virtual RPs of GB and around 5% of virtual RPs of GS when the data is fully sampled. This is because the LS approach first performs the local indoor area partitioning on the training sets and then augments the local areas. Furthermore, it can be observed from Table 4 that the number of RPs generated by LS is even less than about 2% of the GB approach in some cases when the sampling ratio is lower than 40%. Although the augmented data can improve the positioning performance against using only the raw data, the improvement is not as significant as the GB and GS approach. This is because the LS approach partitioned the indoor areas locally, which highly relies on the distribution of the raw data. When the data amount and density are very low, the LS approach will fail to cover the investigated indoor region by missing many areas.

#### 5.2.4. Comparison with State-of-the-Art

In the earlier sections, we thoroughly assessed the performance of the three VRPG algorithms linked with the proposed SWSM in terms of positioning accuracy, floor identification accuracy, and computation complexity. In this section, our focus shifts to evaluating the proposed SWSM’s generalization ability in comparison to the widely used inverse distance weighting (IDW) algorithm, as well as its effect on positioning accuracy. Specifically, we analyze the ability of both methods to generate RSS values on unsurveyed points and how this influences the accuracy of the resulting position estimates.

Inverse Distance Weighting: The IDW algorithm is a commonly used interpolation technique used in geographic information systems (GIS) to estimate values at unsampled locations based on nearby sampled points [35]. It works by assigning a weight to each sampled point based on its distance to the unsampled point, with closer points being given higher weights. The estimated value at the unsampled point is then calculated as a weighted average of the values at the nearby sampled points, where the weights are determined by their distance to the unsampled point. The IDW algorithm assumes that the values being interpolated have a spatial correlation and that closer points have a greater influence on the estimated value than more distant points. The algorithm is widely used in environmental modeling, spatial analysis, and cartography. In this study, we interpolate the RSS values by APs and assemble them as new FPs on each location.

The error of WiFi signal modeling: To compare the generalization ability of IDW and the proposed MGPR, we predicted the RSS values of each AP on the reference points in the test set and calculated the absolute error between the ground-truth RSS and predicted RSS. The RSS values with a default value (−110 dBm ) that denote the non-detected signals have been removed from the test set before calculation.

**Figure 11:**
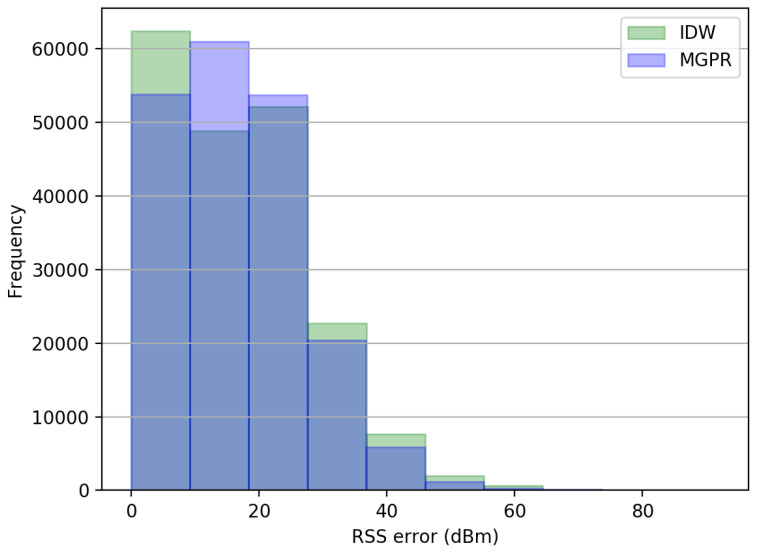
Histogram of the RSS error between the ground-truth and the predictions.

As listed in Table 5, the MGPR shows a similar median error to the IDW but a lower mean and standard deviation in predicting RSS values on unsurveyed locations. In addition, we define an invalid estimation of RSS value if it equals −110 dBm. We can see from the table that IDW provides more invalid RSS predictions than MGPR, which shows a weaker generalization ability. Furthermore, we can observe from the histogram of the RSS prediction errors in Figure 11 that the proposed MGPR has a higher frequency to show a lower RSS estimation error than 30 dBm. Contrastingly, the IDW has a higher chance of gaining an error higher than 30 dBm.

**Figure 12:**
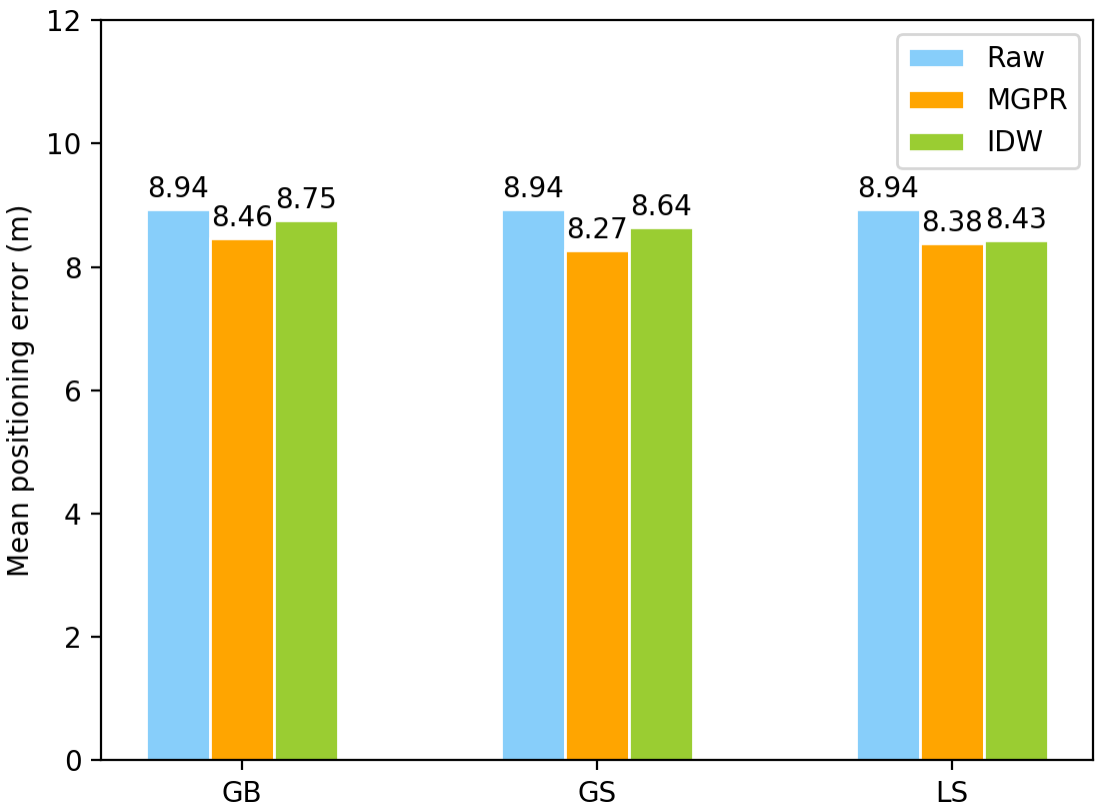
The comparison of mean position accuracy using different algorithms.

Positioning performance: We compared the positioning performance of the IDW and the proposed MGPR on accuracy in positioning and floor identification. We used the entire dataset with a sampling ratio of 1.0 for evaluation. Figure 12 shows that the IDW, with the three VRPG approaches, generally improves positioning accuracy compared to using only raw data. However, in all cases using GB, GS, and LS, MGPR showed lower positioning errors than IDW. This is because the proposed MGPR can capture the global distribution of all WiFi FPs in the 3D environment and generate new FPs that are distinct from each other. This helps the positioning algorithm better identify the user’s position.

**Figure 13:**
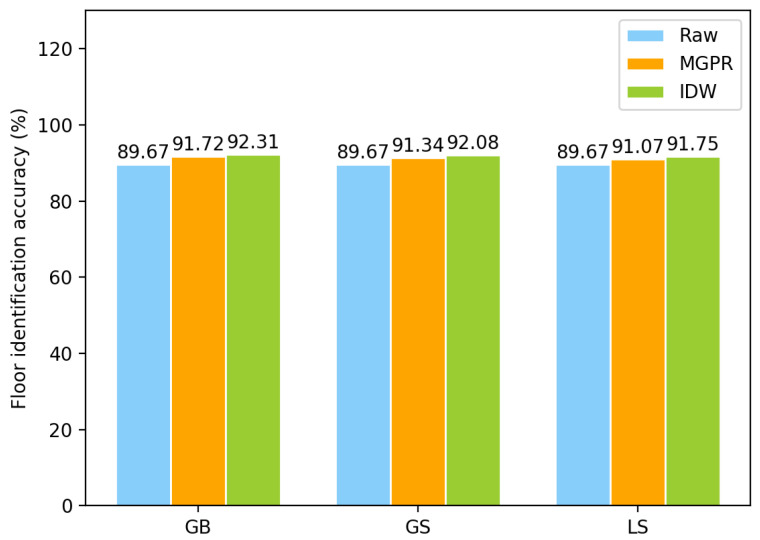
The comparison of floor classification accuracy using different algorithms.

On the other hand, the IDW outperforms MGPR in floor identification accuracy, as illustrated in Figure 13. This suggests that the FPs generated by MGPR have better locality but worse commonalities than those generated by IDW. The reason for this is that MGPR models the distribution of all FPs in the 3D environment and maps the RSS values of each FP to a specific coordinate. In contrast, IDW first interpolates the RSS values with APs and then assembles the new RSS values to new FPs on each coordinate. As a result, for the new FPs generated by IDW, it is easier to identify their commonalities and they perform better in floor identification.

## 6. Conclusions

In this paper, we have proposed a scalable WiFi FP augmentation method for 3D crowdsourced indoor positioning systems in large complex indoor environments. One key element of augmentation with the proposed method is the proposed SWSM algorithm that creates an MGPR model to estimate the joint distribution of all WiFi signals in a 3D environment. Another one is to design two self-adaptive VRPG approaches to reduce the non-necessary virtual RPs from the well-adopted GB approach. With these two elements, we generate more FPs by estimating the RSS values on virtual RPs to augment the training set. Experiments on an open public dataset of a 3D building have shown that the proposed WiFi FP augmentation method can improve positioning accuracy and building identification accuracy even with insufficient data coverage. Furthermore, the two self-adaptive VRPG approaches can provide better improvement in positioning performance than the conventional GB approach. In detail, the GS can significantly improve positioning performance with halved computation complexity than GB; while the LS approach can reduce 90% of the computation complexity of GB and maintain a moderate improvement in positioning performance when there is sufficient data coverage.

However, a comparison between the proposed MGPR model and the widely-adopted IDW algorithm shows that the proposed algorithm can generate FPs with a better locality but worse commonalities. This is attributed to the fact that the MGPR provides higher positioning accuracy but lower floor identification accuracy than IDW, which motivates us to focus on developing floor-based MGPR models in future work, aiming to enhance the commonality of augmented FPs.

## Figures and Tables

**Table 1:** The number of samples (reference points) on each floor in the training and testing set.

Floor *	Train	Test
0	226	1265
3.7	197	1108
7.4	139	770
11.1	118	699
14.8	17	109
Total	697	3951

* Note that the floor number is denoted by the value of the *z*-axis of the samples.

**Table 2:** 3D positioning accuracy evaluations on building-based augmentation *.

Samping Ratio	Raw Data (m)	GB (m)	GS (m)	LS (m)
10%	21.27	**17.21**	18	19.82
20%	14.47	**12.95**	13.06	13.08
30%	13.57	11.22	**10.34**	12.10
40%	13.01	**11.72**	11.76	12.21
50%	11.36	10.60	**10.55**	10.72
60%	10.93	10.24	**9.85**	10.00
70%	10.14	9.17	**9.07**	9.26
80%	9.83	9.36	**8.85**	8.86
90%	9.06	8.59	**8.37**	8.44
100%	8.94	8.46	**8.27**	8.38

* Note that the highest positioning accuracy in each case is marked with bold fonts.

**Table 3:** Floor identification accuracy evaluations on building-based augmentation *.

Sampling Ratio	Raw Data (%)	GB (%)	GS (%)	LS (%)
10%	60.01	66.67	**68.21**	62.90
20%	74.79	74.66	75.53	**75.96**
30%	76.26	**86.46**	85.90	82.49
40%	80.49	81.22	**82.38**	81.60
50%	82.36	82.99	82.97	**83.24**
60%	83.85	**88.10**	87.37	85.50
70%	86.76	89.67	**90.66**	89.95
80%	89.24	91.09	**91.24**	91.07
90%	89.32	91.39	**91.60**	91.27
100%	89.67	**91.72**	91.35	91.07

* Note that the highest floor identification accuracy in each case is marked with bold fonts.

**Table 4:** Comparison of the number of RPs in different cases *.

	Floor 0			Floor 1			Floor 2			Floor 3			Floor 4		
	GB	GS	LS	GB	GS	LS	GB	GS	LS	GB	GS	LS	GB	GS	LS
10%	5850	2935	**272**	6133	3213	**174**	5232	1881	**13**	2533	1311	**50**	2	**1**	**1**
20%	11,016	4915	**427**	7295	3244	**300**	7670	3672	**102**	4779	2852	**167**	3303	1119	**3**
30%	11,411	6038	**662**	8438	4361	**595**	7571	4338	**369**	3427	2436	**204**	3349	207	**7**
40%	9196	5520	**1101**	8463	4925	**656**	10,406	4940	**372**	6116	3685	**261**	666	227	**17**
50%	13,327	6757	**1398**	8748	5083	**851**	7057	4269	**439**	5865	3233	**338**	511	209	**9**
60%	12,363	6628	**1473**	8962	5266	**1224**	10,703	4363	**587**	6270	3937	**489**	3658	1132	**39**
70%	12,197	7209	**1665**	8982	5866	**1265**	8097	5094	**747**	5799	3277	**701**	612	103	**22**
80%	11,044	7292	**1757**	9135	5339	**1329**	10,736	4521	**794**	6561	4112	**630**	693	178	**40**
90%	13,757	7795	**1839**	9154	5439	**1399**	8132	4430	**955**	6576	4231	**723**	3742	1155	**43**
100%	13,787	7903	**2100**	9173	5466	**1501**	10,764	4458	**847**	6586	4019	**760**	3743	1156	**45**

* Note that the lowest number of total RPs in each case is marked with bold fonts.

**Table 5:** Comparison of the error in predicting RSS values using different algorithms.

	IDW	MGPR
Number of invalid RSS predictions	1371	494
Mean error (dBm)	16.83	16.67
Median error (dBm)	16.08	16.08
Standard deviation (dBm)	11.51	10.39

## Data Availability

Data available on request from the authors.

## Abbreviations

The following abbreviations are used in this manuscript:

RFID	Radio frequency identification
RSS	Received signal strength
FP	Fingerprint
RP	Reference point
GPR	Gaussian process regression
MGPR	Multivariate Gaussian process regression
VRPG	Virtual reference point generation
SWSM	Spatial WiFi signal modeling
GB	Grid-based
GS	Globally self-adaptive
LS	Locally self-adaptive
